# Nutritional and Sensory Evaluation of Complementary Foods Developed From Staple Grains and Dried Dagaa Fish (*Stolothrissa tanganicae*)

**DOI:** 10.1002/fsn3.72463

**Published:** 2026-10-12

**Authors:** Marie Amélie Nabuholo, Olouwafemi Mistourath Mama, Adama Diouf, Esto Bahizire, Galibwa Kulimushi Alexis, Ciribagula Nkulwe Rodrigue, Nicole Idohou‐Dossou

**Affiliations:** ^1^ Laboratoire de Recherche en Nutrition et Alimentation Humaine (LARNAH), Département de Biologie Animale, Faculté des Sciences et Techniques Université Cheikh Anta Diop (UCAD) Dakar Senegal; ^2^ Center for Tropical Diseases and Global Health Catholic University of Bukavu Bukavu South Kivu Democratic Republic of Congo; ^3^ Centre de Recherche en Sciences Naturelles (CRSN) Bukavu Democratic Republic of Congo

**Keywords:** complementary food, dagaa fish, food formulation, South Kivu, stunting

## Abstract

Plant‐based complementary foods commonly consumed in South‐Kivu are often deficient in protein and essential micronutrients, contributing to undernutrition among young children. This study developed fish‐enriched complementary flours and evaluated their proximate composition, sensory quality, acceptability, and storability. Three composite flours were formulated: a control complementary flour (CCF, no fish) and two enriched formulations containing 40% (ECF1) and 20% (ECF2) dried dagaa. Proximate composition and mineral content were determined using AOAC methods, and the sensory acceptability of CCF and ECF1 was evaluated among 82 mother–child pairs using a 7‐point hedonic scale. Nutrient composition differed significantly among formulations (*p* < 0.0001). ECF1 had the highest levels of protein (33.1 g/100 g), iron (11.5 mg/100 g), zinc (6.54 mg/100 g), and calcium (820.5 mg/100 g), whereas CCF had the highest carbohydrate (71.7 g/100 g) and energy (408 kcal/100 g) contents. Based on a 50 g dry flour serving as recommended by WFP, ECF1 provided the greatest estimated contribution to protein and mineral requirements for infants aged 6–12 months. Sensory evaluation showed that both mothers and children preferred CCF to ECF1 (*p* < 0.0001). Nevertheless, ECF1 achieved acceptable child acceptability (68.3% with an appreciation index ≥ 70%), and nearly two‐thirds of mothers expressed willingness to offer the product despite its lower sensory scores. Both formulations remained microbiologically safe after 6 months of ambient storage. These findings demonstrate that dried dagaa substantially improves the nutrient density of cereal‐based complementary foods while maintaining acceptable sensory properties and storage stability, supporting its potential as a sustainable food‐based strategy to improve complementary feeding in resource‐limited settings.

## Graphical abstract

Complementary foods in South Kivu, DRC, are predominantly plant‐based and may provide limited amounts of key micronutrients. This study developed three flours: a control (CCF) and two dagaa fish‐enriched formulas (ECF1: 40%; ECF2: 20%) from blends of maize, sorghum, soybean, and dried *Stolothrissa tanganicae* . Fish inclusion improved protein and mineral contents. CCF was more sensorially accepted than ECF1, whereas both formulations showed satisfactory microbiological quality at baseline and after 6 months of storage.

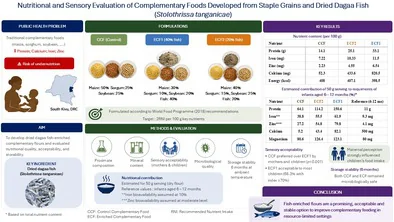

## 1 Introduction

Malnutrition remains a critical public health challenge in the Democratic Republic of Congo (DRC), particularly among infants and young children. In South Kivu, the situation is alarming: 48% of children under five are stunted, 20.3% are underweight, and 3% are wasted, with a mortality rate of 4% (Publique 2020). These figures place South Kivu among the highest‐burden regions in sub‐Saharan Africa, representing a major barrier to sustainable development and progress toward the United Nations Sustainable Development Goals (SDGs). South Kivu was historically an important food‐producing region in the DRC; however, persistent armed conflicts have disrupted livelihoods and agricultural production, reduced access to food, and exacerbated food insecurity and limited dietary diversity (Burns et al. 2016; Kandala et al. 2011). These conditions can lead to an inadequate intake of essential nutrients, especially among young children. Micronutrient deficiencies are a key driver of this burden: approximately 17.3% of the global population has inadequate zinc intake, and nearly 30% suffer from anemia, predominantly due to iron deficiency (Belay et al. 2021; Dahdouh et al. 2019). In low‐income regions such as South‐Kivu, deficiencies are exacerbated by the widespread consumption of nutrient‐poor, cereal‐based complementary foods (Burns et al. 2016). Such diets are often deficient in essential micronutrients, such as iron and zinc, as well as the protein necessary for healthy growth, leading to stunted growth, increased susceptibility to illness, and child mortality (Allen 2012).

Complementary feeding plays a key role in bridging the gap between exclusive breastfeeding and a non‐diversified family diet by providing vital nutrients during a crucial window of growth and development (Lutter and Dewey 2003). From 6 months of age, breast milk alone is insufficient to meet an infant's growing nutritional needs, and the quality of complementary foods determines child health outcomes. Therefore, adopting high‐quality complementary foods is essential for the proper nutrition of infants and young children (de Pee and Bloem 2009). However, in most developing countries, including DRC, complementary foods are predominantly plant‐ and cereal‐based and have low nutrient and energy density (Mouafo et al. 2024; Van der Merwe et al. 2019; Burns et al. 2016).

Evidence shows that children whose diets include animal‐source foods grow better than those on exclusively plant‐based diets (Allen 2012). Among animal‐source foods, fish, particularly small, whole‐dried species, are highly nutritious and cost‐effective. Consumed whole, they are exceptionally rich in iron, zinc, calcium, and essential omega‐3 fatty acids, often exceeding other animal‐source foods in micronutrient density (Thilsted et al. 2016). When dried and ground into powder, small fish products provide levels of iron, zinc, and calcium comparable to those in commercially produced micronutrient supplements (Byrd et al. 2021). In addition, small fish species typically have high reproductive rates and are harvested below their maximum sustainable yield in several regions of sub‐Saharan Africa (Kolding et al. 2019). These attributes make small fish, such as dagaa, particularly suitable for inclusion in interventions aimed at reducing malnutrition in vulnerable populations. Previous studies in sub‐Saharan Africa have demonstrated the efficacy of incorporating small, dried fish into complementary food formulations to increase dietary intakes of iron, zinc, and calcium (Gibson et al. 2003; Lartey et al. 1999). In Malawi, maize‐soybean‐fish composite foods based on *Engraulicypris sardella* (usipa) have shown promising results in improving the nutritional quality and sensory acceptability of complementary foods for infants aged 6–23 months (Msiska et al. 2023; Kaunda et al. 2025). Similar approaches using small fish species have been reported in Ghana, Ethiopia, Tanzania, and Zambia (Affonfere et al. 2021; Mekuria et al. 2021; Tedom Dzusuo et al. 2024; Ragsdale et al. 2026). However, the formulation of complementary foods incorporating locally available fish species from the Great Lakes region—particularly dagaa ( *Stolothrissa tanganicae* ), a small fish endemic to Lake Tanganyika and widely harvested in South Kivu—remains largely unexplored.

A preliminary study in two health zones of South Kivu identified the food items most commonly used as complementary foods for children aged 6–23 months (Nabuholo et al. 2025). Maize, sorghum, and soybean flours were the predominant components, confirming the plant‐based and micronutrient‐poor nature of local complementary feeding practices.

Although home‐based fortification is recognized as an effective strategy to improve nutrient intake during complementary feeding (Mekuria et al. 2021) and is prioritized in the DRC's 2023–2030 Strategic Nutrition Plan, fortified composite flours for young children remain insufficiently promoted.

Dagaa is a locally available, affordable, year‐round accessible animal‐source food with high potential to enrich these formulations. To the authors' knowledge, no study has systematically evaluated the incorporation of dagaa into complementary food formulations using a linear programming approach applied to locally available staple ingredients, nor has any study combined nutritional characterization, microbiological safety assessment, and sensory acceptability evaluation in a single study in this context.

In this context, the present study aimed to develop complementary food flours from blends of staple cereals and dried dagaa fish (*S. tanganicae*) and to evaluate their nutritional composition and sensory acceptability, with roasting as the primary processing method.

## 2 Materials and Methods

### 2.1 Ingredients Procurement and Preparation

The local plant‐based ingredients selected for the complementary food formulation were white maize ( *Zea mays* L.), red sorghum ( *Sorghum bicolor* ), and soybean ( *Glycine max* ) seeds. These ingredients were chosen as the primary cereal components because they are the most widely cultivated and consumed staple grains in the region (Nabuholo et al. 2025). Soybean was included as a complementary source of protein and energy.

Dried dagaa fish (*S. tanganicae*) was selected as the animal‐source ingredient for its local availability, affordability, year‐round accessibility in South Kivu, and high micronutrient density.

This experiment aimed to improve locally available complementary foods by incorporating affordable animal‐source ingredients to enhance their nutritional quality. White maize (25 kg), sorghum (12 kg), soybean (15 kg), and dried dagaa fish (10 kg) were purchased from the local market in Bukavu, South Kivu, packaged separately, and transported to the Olame Center within 30 min. Upon arrival, the ingredients were stored under appropriate conditions for 24 h before processing. Proximate and mineral analyses were conducted on both the raw ingredients and the formulated complementary flours. Moisture, ash, protein, lipid, carbohydrate, and energy contents were determined in duplicate, whereas calcium (Ca), iron (Fe), magnesium (Mg), and zinc (Zn) concentrations were measured in triplicate.

The production of the complementary flours followed a series of standardized processing steps (Figure 1).

**FIGURE 1:**
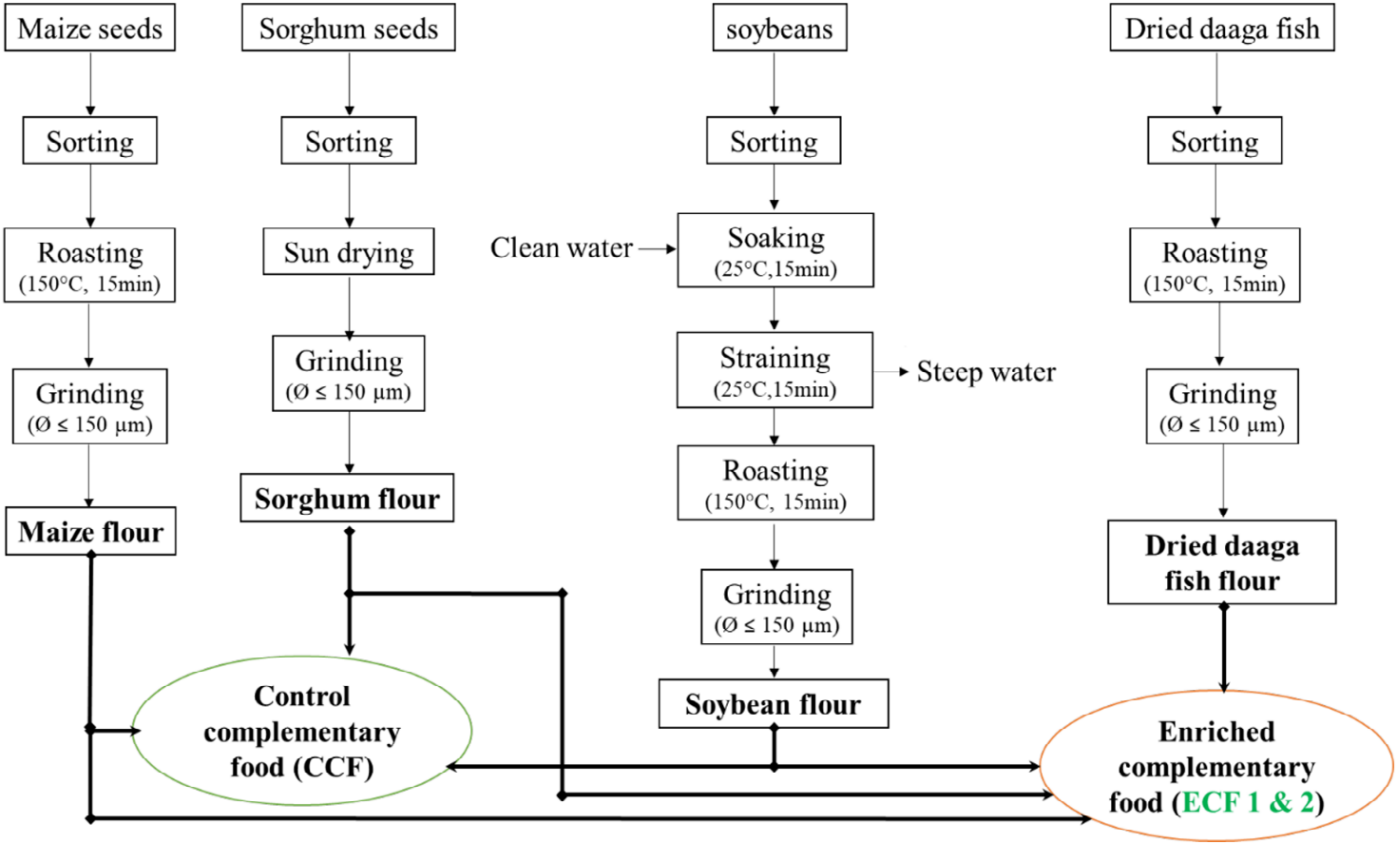
Flow chart of sample processing and formulation of complementary food products. CCF: 50% maize, 25% sorghum and 25% soya flour; ECF1: 30% maize, 10% sorghum, 20% soybean flour and 40% dried dagaa fish flour; ECF2: 40% maize, 15% sorghum, 25% soybean flour and 20% dried dagaa fish flour.

White maize grains were first sorted to remove stones, foreign materials, insects, and damaged kernels. The cleaned grains were then roasted in a locally manufactured roaster at 200°C for 30 min until a characteristic brown color developed, following traditional African food processing practices (Agume Ntso et al. 2017). After cooling to room temperature, the roasted grains were milled using a hammer mill with a 150 μm sieve to obtain maize flour.

Sorghum grains underwent a similar cleaning process to remove stones, foreign materials, insects, and defective grains. Unlike maize, the cleaned sorghum grains were milled directly, without roasting, and then sieved through a 150 μm mesh to produce sorghum flour.

Soybeans were sorted to remove impurities, damaged seeds, and insects, then soaked in water for 15 min to soften the beans and reduce antinutritional factors. The soaked beans were drained and roasted at 200°C for 30 min. This heat treatment was intended to inactivate antinutritional compounds, particularly trypsin inhibitors, and to improve the flour's sensory characteristics. After cooling, the roasted soybeans were milled and sieved through a 150 μm sieve to obtain soybean flour.

Dried dagaa fish were cleaned by removing stones and other foreign matter, then roasted at 150°C for 15 min to reduce microbial contamination and minimize undesirable odors. The roasted fish were subsequently cooled, ground, and sieved through a 200 μm mesh to produce fish meal.

Finally, all flours were individually packaged in clean, labeled polyethylene bags and stored at room temperature until formulation and subsequent analyses.

### 2.2 Formulation of Complementary Foods

Three composite flours were formulated using different ingredient ratios (Figure 1). The quantities of each ingredient in the Enriched Complementary Food (ECF) formulation were determined using linear programming and the Solver‐Simplex LP function in Microsoft Excel as described by (Chakeredza et al. 2008). The primary objective was to minimize the cost of the ECF formulation while ensuring nutritional adequacy. The equation system used is as follows:

$$a_{1}X_{\text{maize}}+a_{1}X_{\text{sorghum}}+a_{1}X_{\text{soybean}}+a_{1}X_{\text{fish}}=b_{1}$$

$$a_{2}X_{\text{maize}}+a_{2}X_{\text{sorghum}}+a_{2}X_{\text{soybean}}+a_{2}X_{\text{fish}}=b_{2}$$

$$a_{n}X_{\text{maize}}+a_{n}X_{\text{sorghum}}+a_{n}X_{\text{soybean}}+a_{n}X_{\text{fish}}=b_{n}$$

where *a* = the nutrient content (carbohydrate, protein, lipid, and energy). *X* = the proportions of the ingredients to be mixed. *b* = the requirements to be met (Table 1).

**TABLE 1:** Nutritional constraints applied to the formulation of enriched complementary flour.

Nutrient	Target (2 × RNI/100 g DM)
Energy (kcal/100 g DM)	420
Protein (g/100 g DM)	16
Added sugar (g/100 g DM)	5
Lipids (g/100 g DM)	10
Calcium (mg/100 g DM)	800
Magnesium (mg/100 g DM)	108
Iron (mg/100 g DM)	23
Zinc (mg/100 g DM)	8.4

*Note:* 2 RNI/100 g: nutrient target corresponding to two times the recommended nutrient intake per 100 g of complementary food, according to WFP (2018).

Abbreviations: DM, dry matter; RNI, recommended nutrient intake.

The function to be optimized, Y, is the objective function. The decision variables, X, are systematically varied to achieve the optimal Y, whereas c denotes the price per gram of each ingredient.

$$\mathrm{Minimize}\;Y=c_{\text{maize}}X_{\text{maize}}+c_{\text{sorghum}}X_{\text{sorghum}}+c_{\text{soybean}}X_{\text{soybean}}+c_{\text{fish}}X_{\text{fish}}$$

The inclusion levels of each ingredient were in accordance with the Nutritional Guidance for Complementary Food, recommended by WFP (2018), per 100 g of flour.

The inclusion levels of each ingredient were constrained according to the WFP (2018) nutritional guidelines for complementary foods, targeting nutrient levels equivalent to two times the recommended nutrient intake (2 RNI) per 100 g of flour. This formulation target is recommended in settings where daily consumption of 50 g of complementary food is unlikely, as it provides approximately one RNI in a 50 g serving and about 50% of the RNI in a more realistic 25 g serving. It is particularly appropriate for populations with large nutrient gaps and a high prevalence of undernutrition, such as children aged 6–23 months in South Kivu (Nabuholo et al. 2025).

Minimum inclusion thresholds were set as follows: 5% for fish flour, 10% for sorghum flour, and 20% for each of maize and soybean flours, based on their respective contributions to the final formulation's nutrient profile.

The first formulation, Control Complementary Food (CCF), consisted solely of vegetable ingredients—maize, sorghum, and soybean flour—in a 50:25:25 ratio. The second formulation, Enriched Complementary Food (ECF), included maize, sorghum, soybean flour, and dried dagaa fish flour in a ratio of 30:10:20:40 for ECF1 and 40:15:25:20 for ECF2.

### 2.3 Proximate Analysis of Raw Ingredient Flours and Roasted Complementary Foods

Samples were transported to the Centre National de Technologie Alimentaire (CNTA) and the Institut des Sciences Agronomiques du Burundi (ISABU), in Burundi, for laboratory analyses. The proximate composition of the raw ingredients and formulated complementary flours was determined according to the standard methods of the Association of Official Analytical Chemists (AOAC 2019). For each analysis, 5 g of dried sample was accurately weighed on an analytical balance (Sartorius BC210S, Göttingen, Germany).

Moisture content was determined by oven drying at 105°C for 2 h (AOAC 930.04). Ash content was measured by incineration in a muffle furnace at 550°C for 8 h (AOAC 930.05), followed by cooling in a desiccator at room temperature. Crude protein was quantified using the Kjeldahl method (AOAC 930.09), and protein content was calculated by multiplying the total nitrogen content by a conversion factor of 6.25 (Mekuria et al. 2021; Kaunda et al. 2025). Crude lipid content was determined by Soxhlet extraction with hexane as the extraction solvent (AOAC 930.09). Total carbohydrate content was calculated by difference [100 − (moisture + ash + protein + lipid)], whereas metabolizable energy was determined using Atwater's calorie conversion factors of 4 kcal/g for crude protein, 9 kcal/g for crude fat, and 4 kcal/g for available carbohydrate (FAO 2003; WHO 1985, 1998).

Mineral composition, including calcium (Ca), iron (Fe), magnesium (Mg), and zinc (Zn), was determined by flame atomic absorption spectrophotometry (Shimadzu AA‐6200; PerkinElmer Analyst 200) following Pinta (1980). Briefly, samples were incinerated at 550°C for 8 h. The resulting ash was dissolved in 20% hydrochloric acid (HCl), filtered through ashless Whatman filter paper, and diluted to 50 mL with distilled water before analysis. Mineral concentrations were expressed as mg/100 g dry matter.

To estimate the nutritional contribution of each developed complementary flour, calculations were performed for a 50 g serving of dry flour, using nutrient reference values for infants aged 6–12 months (FAO/WHO 2004). Percentage contributions were calculated by dividing the nutrient content in a 50 g serving by the corresponding FAO/WHO RNI for infants and young children. Iron requirements were assumed to have 10% bioavailability, whereas zinc requirements were based on moderate zinc bioavailability. A 50 g serving size was selected because it aligns with the WFP (2018) formulation guidelines for complementary foods and reflects a realistic daily intake.

### 2.4 Mother–Child Pairs Accept Complementary Foods

#### 2.4.1 Study Design, Area, Recruitment, and Ethical Approval

A cross‐sectional study was conducted to assess the sensory acceptability of the developed complementary foods among mother–child pairs in the Miti‐Murhesa Health Zone, South Kivu Province, Democratic Republic of Congo, where maize‐, sorghum‐, and soybean‐based porridges are commonly used for infant and young child feeding. According to the International Organization for Standardization (ISO 2017), a minimum of 70 participants is required to assess the degree of liking (DOL) using a 7‐point hedonic scale with 80% power and a 5% significance level. Accordingly, 82 mother–child pairs with children aged 6–23 months were randomly selected from vaccination registers.

Eligible mothers received study information and provided oral informed consent before enrolment. Trained research assistants screened participants for eligibility. Children were included if they were aged 6–23 months, appeared healthy on the day of assessment, had a weight‐for‐length *z*‐score (WLZ) > −2, and were able to consume porridge as confirmed by their mothers. Mother–child pairs were excluded if the child was ill or had a known or suspected intolerance or allergy to fish.

The study protocol was approved by the Ethics Committee of the Catholic University of Bukavu (number UCB/CIES/NC/014/2023). Administrative authorization to conduct the study was also obtained from the Provincial Health Division (Division Provinciale de la Santé, DPS) of South Kivu.

#### 2.4.2 Sensory Analysis

Sensory evaluation was conducted to assess the acceptability of the developed complementary food porridges among mothers and their children. The porridges were prepared by mixing 4000 g of each composite flour with 16,000 mL of water and cooking the mixture for approximately 15 min until a semi‐solid consistency was achieved, at which point the porridge could be easily detached from the spoon. The porridges were then cooled to approximately 45°C before serving.

Trained research assistants conducted the evaluation at an appropriate testing site over two consecutive days, involving 82 mother–child pairs. To minimize order bias, the sequence in which the porridges were presented was reversed on the second day. Each mother received 200 g of each porridge but was not required to consume the entire serving. Mothers evaluated the color, aroma, taste, texture, and overall acceptability of each porridge using a 7‐point hedonic scale ranging from 1 (“dislike very much”) to 7 (“like very much”), with a score of 4 indicating “neither like nor dislike” (Figure 2) (Tomlins et al. 2007; Badiane et al. 2018). To reduce carryover effects, mothers were instructed to rinse their mouths with clean water between samples, and an 180‐min interval was maintained between the two tasting sessions.

**FIGURE 2:**
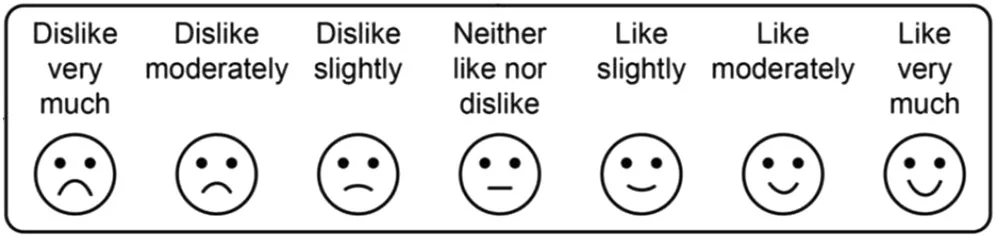
Seven‐point facial hedonic scale (Tomlins et al. 2007).

Direct sensory evaluation in children aged 6–23 months is challenging because of their limited cognitive development, short attention spans, and an inability to provide reliable verbal responses (Kaunda et al. 2025; Badiane et al. 2018). Therefore, child acceptability was assessed indirectly through maternal observations supported by trained research assistants. Each child was offered a 200 g portion of the test porridge approximately 60 min after the last breastfeeding session. Mothers were instructed to feed their children until the entire portion was consumed or until the child refused further feeding, whereas research assistants recorded the child's facial expressions and feeding behavior throughout the evaluation. Mothers subsequently rated their child's overall acceptability of each porridge using the same 7‐point hedonic scale and were invited to provide additional comments on the child's reactions.

The amount of porridge consumed was determined by weighing the remaining portion on a digital bowl scale (iBalance 5000g, GKS46513, GEEPAS), and the consumption time was recorded for each child. Food intake was then classified using a 5‐point scale: 1 = complete refusal (0% consumed); 2 = less than one‐quarter consumed; 3 = approximately half consumed; 4 = more than three‐quarters consumed; and 5 = entire portion consumed (200 g).

The index of acceptability (IA) for both mothers and their children was calculated by using the equation:

$$\mathrm{IA}\;\left(\%\right)=\frac{\text{hedonic score}*100}{7}$$

where the hedonic score represented the overall acceptability rating reported by the mother. Food should have a minimum score of 70% IA to be considered acceptable (Tomlins et al. 2007; Badiane et al. 2018).

This approach enabled the assessment of both maternal and child acceptability, which is a critical determinant of the adoption and sustained use of novel complementary foods in target communities.

### 2.5 Storage Stability of the Composite Flour

The storage stability of the enriched complementary flour (ECF1) and the control complementary flour (CCF) was evaluated under ambient conditions over 6 months. To simulate typical household storage practices, each flour was packaged in 500 g portions in transparent, food‐grade polyethylene bags and sealed with a double heat seal. The sealed packages were placed in cardboard boxes and stored in a well‐ventilated room at the Centre Olame at ambient temperature representative of local household storage. A 6‐month storage period was selected because it is widely used to assess the stability, shelf life, and quality of food products under normal storage conditions, allowing the monitoring of physicochemical, microbiological, and sensory changes that may occur over time (Amit et al. 2017). Samples were collected at baseline (M0) and at monthly intervals throughout the storage period, then transported to the Centre National de Technologie Alimentaire (CNTA) and the Institut des Sciences Agronomiques du Burundi (ISABU), in Burundi, for laboratory analyses. Proximate composition was determined using the analytical procedures described in Section 2.2. Changes in moisture, protein, lipid and mineral contents were monitored to evaluate physicochemical stability during storage. Microbiological analyses were performed by the CNTA to evaluate the safety and quality of the flours in accordance with the microbiological specifications for cookable complementary flours. The analyses included detection of fecal coliforms, *Escherichia coli* , and *Salmonella* spp. according to ISO 4832, ISO 7251, and ISO 6785, respectively. Yeasts and molds were enumerated according to ISO 21527. These assessments were conducted at baseline (M0) and after 6 months of storage (M6) to verify compliance with microbiological safety standards for complementary foods intended for infants and young children.

### 2.6 Statistical Analysis

Statistical analyses were conducted in RStudio (R version 4.4.3; RStudio Inc., Boston, MA, USA) and Stata/SE version 16 (StataCorp, College Station, TX, USA). Normality and homogeneity of variances were assessed using the Shapiro–Wilk and Bartlett's tests, respectively. Results are presented as means ± standard deviations (SD). For nutritional composition data, because the assumption of homogeneity of variances was not met, differences among formulations were assessed using the Kruskal–Wallis test followed by Dunn's post hoc test with Bonferroni adjustment. A one‐way ANOVA with Bonferroni‐adjusted pairwise comparisons were also performed as a sensitivity analysis to assess the robustness of the findings. Sensory evaluation data were analyzed using paired Wilcoxon signed‐rank tests. Storage stability data were summarized descriptively by comparing nutritional and microbiological characteristics at baseline and after 6 months of storage. Factors associated with porridge consumption were examined using separate multivariable linear regression models for CCF and ECF1. The dependent variable was the child's intake score on a 5‐point scale. Independent variables included the child's behavioral state at feeding (awake, calm, or crying) and the mother's willingness to offer the porridge based on her acceptability rating. Regression results are reported as coefficients (*β*), standard errors (SE), *t*‐statistics, and *p*‐values. Statistical significance was set at *p* < 0.05.

## 3 Results

### 3.1 Proximate Composition of Raw Ingredients

The proximate and mineral composition of the raw ingredients (dry weight basis) is presented in Table 2. Moisture content ranged from 3.14 to 13.00 g/100 g, with sorghum showing the highest value and soybeans the lowest (*p* < 0.05). Significant differences in protein content were observed among the ingredients (*p* < 0.001). Dried dagaa fish was the richest protein source (72.1 ± 0.14 g/100 g), followed by soybean (35.1 ± 2.97 g/100 g), whereas maize and sorghum contained considerably lower protein levels (9.71 ± 0.15 and 10.08 ± 1.17 g/100 g, respectively). Lipid content also varied significantly, with soybean containing the highest concentration (21.1 ± 0.14 g/100 g), followed by dried dagaa fish, maize, and sorghum.

**TABLE 2:** Nutrient composition of individual ingredients on a dry weight basis per 100 g.

Nutrients	Maize	Sorghum	Soybean	Dried dagaa fish
Moisture content, %	3.8 ± 0.2^a^	13 ± 0^b^	3.14 ± 0.01^a^	3.2 ± 0.2^a^
Energy, kcal	402.2 ± 1.1^a^	355.6 ± 5.7^b^	471.6 ± 0.8^c^	366 ± 6.2^b^
Carbohydrates, g	80.4 ± 0.02^a^	72.3 ± 0.00^a^	35.33 ± 3.1^b^	7.05 ± 1.2^c^
Crude protein, g	9.71 ± 0.15^a^	10.08 ± 1.17^a^	35.1 ± 2.97^b^	72.1 ± 0.14^c^
Lipid, g	4.65 ± 0.08^a^	2.9 ± 1.13^a^	21.1 ± 0.14^b^	5.5 ± 1.28^c^
Ash, g	1.4 ± 0.01^a^	1.7 ± 0.02^a^	5.3 ± 0.05^b^	12.2 ± 0.3^c^
Iron, mg	3.78 ± 0.59^a^	13.85 ± 0.64^b^	10.65 ± 0.07^c^	18.55 ± 0.07^b^
Zinc, mg	1.86 ± 0.07^a^	2.07 ± 0.11^a^	4.31 ± 0.06^b^	19.05 ± 0.07^c^
Calcium, mg	14.54 ± 3.39^a^	17.22 ± 0.83^a^	223 ± 15.41^b^	855.9 ± 0.00^c^
Magnesium, mg	143.5 ± 9.19^a^	166.2 ± 3.5^a^	293.7 ± 3.54^b^	188.7 ± 6.01^c^

*Note:* Data are means ± standard deviation. Within a row, means with different superscripts (letters) differ significantly (*p* < 0.05). The standard deviation of zero reflects two identical replicate measurements.

Carbohydrate content differed markedly among the ingredients (*p* < 0.001). Maize contained the highest proportion of carbohydrates (80.4%), followed by sorghum (72.3%), whereas soybeans had an intermediate content (35.3%). Dried dagaa fish contained only a small amount of carbohydrates (7.05%). The carbohydrate content of maize and sorghum differed only marginally (*p* = 0.050), whereas both were significantly higher than that of soybeans and fish.

The mineral composition also varied significantly across ingredients. Dried dagaa fish was the richest source of iron (18.55 ± 0.07 mg/100 g), followed by sorghum and soybean, whereas maize contained the lowest concentration. Similarly, dried dagaa fish had the highest zinc content (19.05 ± 0.07 mg/100 g), significantly exceeding those in soybeans, sorghum, and maize (*p* < 0.05). No significant difference in zinc concentration was detected between maize and sorghum (*p* = 0.369).

Marked differences were also observed in calcium and magnesium contents. Dried dagaa fish contained exceptionally high calcium levels (855.9 ± 00 mg/100 g), nearly four times higher than soybean and substantially greater than those of maize and sorghum. In contrast, soybeans were the richest source of magnesium (293.7 ± 3.54 mg/100 g), followed by dried dagaa fish, sorghum, and maize (*p* < 0.001).

### 3.2 Nutritional Composition of Composite Flours and Their Contribution to FAO/WHO Recommended Nutrient Intakes (RNI)

The proximate and mineral composition of the formulated complementary flours (dry weight basis) is presented in Table 3. Significant differences were observed among the formulations for all nutrients analyzed (*p* < 0.05). Moisture content ranged from 3.85 to 4.98 g/100 g, with the control complementary flour (CCF) exhibiting the highest value, followed by ECF2 and ECF1. The lower moisture content of the enriched formulations may improve storage stability. Energy values differed significantly among the formulations (*p* < 0.05). CCF had the highest energy content (407.7 kcal/100 g), followed by ECF2 (407.1 kcal/100 g) and ECF1 (398.5 kcal/100 g). Although the energy content of ECF1 was slightly below the WFP (2018) target nutrient level (2 RNI/100 g) of 420 kcal/100 g, it provided approximately 95% of the recommended value, whereas both CCF and ECF2 provided 97% and 96.9%, respectively. Marked improvements in macronutrient and mineral composition were observed following enrichment with dried dagaa fish. Protein content increased significantly from 14.1 g/100 g in CCF to 25.1 g/100 g in ECF2 and 33.1 g/100 g in ECF1 (*p* < 0.001). Conversely, carbohydrate content decreased progressively from 71.7% in CCF to 50.4% in ECF1 (*p* < 0.001), reflecting the replacement of carbohydrate‐rich ingredients with protein‐rich raw materials. Lipid content is below the WFP's recommendation for all formulations.

**TABLE 3:** Nutrient content of complementary flour produced on a dry weight basis per 100 g.

Nutrients	CCF	ECF1	ECF2	2 RNI/100 g^1^
Moisture content, %	4.98 ± 0.32^a^	3.85 ± 0.11^b^	4.27 ± 0.11^a^	< 10^2^
Energy, kcal	408 ± 0.89^a^	398 ± 0.40^b^	407.07 ± 0.42^a^	420
Crude protein, g	14.1 ± 0^a^	33.1 ± 0.06^b^	25.13 ± 0.65^b^	16
Carbohydrates, g	71.7 ± 0.36^a^	50.4 ± 0.2^b^	59.26 ± 0.55^b^	
Fat, g	7.16 ± 0.06^a^	7.16 ± 0.05^a^	7.76 ± 0.05^b^	10
Ash, g	2.03 ± 0.07^a^	5.48 ± 0.05^b^	3.68 ± 0.05^b^	
Iron, mg	7.22 ± 0.04^a^	11.5 ± 0.15^b^	10.33 ± 0.21^c^	23
Zinc, mg	2.23 ± 0.09^a^	6.54 ± 0.75^b^	4.55 ± 0.09^c^	8.3
Calcium, mg	52.3 ± 1.82^a^	820.5 ± 13.30^b^	433.6 ± 9.61^c^	800
Magnesium, mg	129 ± 1.36^a^	197 ± 1.01^b^	202.2 ± 1.61^c^	108

*Note:* Data are means ± standard deviation. Within a row, means with different superscripts (letters) differ significantly (*p* < 0.05). CCF: 50% maize, 25% sorghum and 25% soyabeans flour; ECF1: 30% maize, 10% sorghum, 20% soybean flour and 40% dried dagaa fish flour; ECF2: 40% maize, 15% sorghum, 25% soybean flour and 20% dried dagaa fish flour.

^1^ Codex alimentarius commission.

^2^ 2 RNI/100 g: nutrient target corresponding to two times the recommended nutrient intake per 100 g of complementary food, according to WFP (2018).

Ash content, an indicator of total mineral content, increased significantly in the enriched formulations, ranging from 3.69 to 5.48 g/100 g, compared with 2.03 g/100 g in CCF (*p* < 0.001), indicating the contribution of dried dagaa fish to the mineral composition.

The mineral composition differed significantly across the formulations, with enriched flours containing higher concentrations of iron, zinc, calcium, and magnesium than the control (all *p* < 0.001). Iron concentration increased from 7.22 ± 0.04 mg/100 g in the control complementary flour (CCF) to 10.33 ± 0.21 mg/100 g in ECF2 and 11.53 ± 0.15 mg/100 g in ECF1. Similarly, zinc content increased from 2.23 ± 0.09 mg/100 g in CCF to 4.55 ± 0.09 mg/100 g in ECF2 and 6.54 ± 0.75 mg/100 g in ECF1. Compared with the WFP (2018) target nutrient levels (2 RNI/100 g), ECF1 provided approximately 50% of the target iron concentration and 79% of the target zinc concentration, whereas ECF2 supplied about 45% and 55%, respectively. In contrast, CCF contributed less than one‐third of the target zinc level and approximately one‐third of the target iron level. Calcium content increased markedly following fish enrichment, from 52.3 ± 1.82 mg/100 g in CCF to 433.6 ± 9.61 mg/100 g in ECF2 and 820.5 ± 13.30 mg/100 g in ECF1. ECF1 slightly exceeded the WFP target of 800 mg/100 g, whereas ECF2 reached approximately 54% of this value. Magnesium content also differed significantly across formulations, increasing from 129.0 ± 1.36 mg/100 g in CCF to 197.0 ± 1.01 mg/100 g in ECF1 and 202.2 ± 1.61 mg/100 g in ECF2. Both enriched formulations exceeded the WFP target of 108 mg/100 g.

Table 4 presents the estimated contribution of a 50 g serving of the developed complementary flours to nutrient requirements. Based on nutrient requirements for infants aged 6–12 months, a 50 g serving of the developed complementary flours showed marked differences in nutritional contribution across formulations.

**TABLE 4:** Estimated contribution of a 50 g serving of complementary flours to nutrient requirements of infants aged 6–12 months.

Nutrients	RNI^a^	CCF, %	ECF1, %	ECF2, %
Protein (g)	11	64.1	150.6	114.2
Iron (mg)	9.3	38.8	61.9	55.5
Zinc (mg)	4.1	27.2	79.8	54.8
Calcium (mg)	500	5.2	82.1	43.4
Magnesium (mg)	80	80.6	123.1	126.4

*Note:* Data are means ± standard deviation. CCF: 50% maize, 25% sorghum and 25% soybean flour; ECF1: 30% maize, 10% sorghum, 20% soybean flour and 40% dried dagaa fish flour; ECF2: 40% maize, 15% sorghum, 25% soybean flour and 20% dried dagaa fish flour.

Abbreviation: RNI, recommended nutrient intake.

^a^ Reference values correspond to nutrient requirements for infants aged 6–12 months. Iron requirements were estimated assuming 10% bioavailability, and zinc requirements were based on moderate zinc bioavailability. Values represent the estimated contribution from total nutrient content provided by a 50 g serving of dry flour.

ECF1 provided the highest contribution, supplying approximately 151% of protein requirements, 62% of iron requirements, 80% of zinc requirements, 82% of calcium requirements, and 123% of magnesium requirements. ECF2 also showed improved nutritional performance compared with CCF, particularly for protein (114%) and magnesium (126%), whereas providing moderate contributions to iron (56%), zinc (55%), and calcium (43%).

In contrast, CCF showed substantially lower contributions of key nutrients, particularly iron (39%), zinc (27%), and calcium (5%). These findings demonstrate the nutritional advantage of incorporating dried dagaa into complementary cereal‐legume foods.

### 3.3 Acceptability of Complementary Foods

#### 3.3.1 Demographic and Anthropometric Data of Participants

Eighty‐two (82) child/mother pairs were recruited for the study with a sex ratio of 1.05 for infants/children. Descriptive statistics for anthropometric measures are presented in Table 5. Of the enrolled mothers, 54% were aged 15–30 years and 46% were aged over 30 years. The mean age of participating infants was 14.9 months, with 36% aged 6–12 months and 63% aged 13–23 months. Girls had a slightly lower LAZ than boys (−1.77 and −1.60, *p* = 0.8), respectively, though none were stunted (LAZ < −2). None of the participating infants were wasted (WLZ < −2), and the average WLZ did not differ significantly between boys and girls (*p* < 0.05). All participating infants were already consuming complementary foods, and among them, 75.9% continued to breastfeed for 12–23 months, with a significant difference between girls and boys (46.6% and 29.3%, respectively; *p* = 0.009). In addition, only 29% of children achieved the minimum dietary diversity, and only 12% achieved the minimum acceptable diet, with no significant difference between girls and boys.

**TABLE 5:** Demographic, anthropometric and IYCF profile of the study population.

	Girls (*n* = 42)	Boys (*n* = 40)	Overall (82)	*p* ^a^
Age (months)	14.7 ± 4.7	15.1 ± 5.2	14.9 ± 4.9	0.7
Length (cm)	40.60 ± 3.18	42.23 ± 3.49	41.40 ± 3.41	0.021
Weight (kg)	8.58 ± 1.21	9.42 ± 1.67	8.99 ± 1.51	0.012
WAZ	−1.01 ± 1.06	−0.93 ± 1.52	−0.97 ± 1.30	> 0.9
LAZ	−1.77 ± 1.28	−1.60 ± 1.90	−1.68 ± 1.61	0.8
WLZ	−0.14 ± 1.03	−0.14 ± 1.34	−0.14 ± 1.18	0.4
Consuming complementary foods (%)	100	100	100	
Continued breastfeeding 12–23 months (%)	46.6	29.3	75.9	0.009
Minimum dietary diversity 6–23 months (%)	17.1	12.2	29.3	0.4
Minimum acceptable diet 6–23 months (%)	6.1	6.1	12.2	> 0.9

*Note:* Data are means ± standard deviation.

Abbreviations: LAZ, height/length for age *z*‐score; WAZ, weight for age *z*‐score; WLZ, weight for height/length *z*‐score.

^a^ Pearson's Chi‐squared test; Wilcoxon rank sum test.

#### 3.3.2 Sensory Analysis and Estimating the Portion of Porridge Consumed

The sensory evaluation and acceptability results for the control complementary flour (CCF) and the enriched complementary flour (ECF1) are shown in Table 6. Overall, both mothers and children showed a significantly greater preference for CCF than for ECF1 (*p* < 0.05).

**TABLE 6:** Sensory analysis of enriched complementary food compared with control complementary food.

	CCF	ECF1	*p* ^a^
*Children (mother‐proxied behavioral ratings)*			
Overall acceptability	6.1 ± 1.8	5.42 ± 2.1	0.0006
AI (%)	86.7	74.7	0.0004
IA ≥ 70%	82.9	68.3	0.0004
Acceptability‐based offer (%)	89.2	68.7	0.0005
*Mothers*			
Color	6.9 ± 0.4	4 ± 2	< 0.0001
Aroma	6.8 ± 0.8	3.5 ± 1.9	< 0.0001
Texture	6.8 ± 0.8	3.6 ± 1.9	< 0.0001
Flavor	6.8 ± 0.9	3.6 ± 1.9	< 0.0001
Overall acceptability	6.8 ± 0.7	3.6 ± 1.9	< 0.0001
AI (%)	96.3	31.7	< 0.0001
IA ≥ 70%	96.3	31.7	< 0.0001
Acceptability‐based offer (%)	91.6	65.1	< 0.0001

*Note:* Data are means ± standard deviation. Within a row, means with different superscripts (letters) differ significantly (*p* < 0.05). CCF: 50% maize, 25% sorghum and 25% soybean flour; ECF1: 30% maize, 10% sorghum, 20% soybean flour and 40% dried dagaa fish flour; acceptability‐based offer: the proportion of mothers willing to offer porridge based on both their own and their child's acceptability.

Abbreviation: AI, appreciation index.

^a^ Pearson's Chi‐squared test; Wilcoxon rank sum test.

Among children's reactions, CCF achieved a significantly higher overall acceptability score than ECF1 (6.07 vs. 5.42 on the 7‐point hedonic scale), indicating that the control formulation was more readily accepted. Although children's preferences for both porridges were generally positive, the lower scores for ECF1 suggest that enrichment with dried dagaa fish slightly reduced product acceptability. The higher preference for CCF was further reflected in the Acceptability Index (AI), with 82.9% of children rating CCF above the acceptability threshold (AI ≥ 70%), compared with 68.3% for ECF1. Likewise, more mothers reported they would be willing to offer or purchase CCF for their children (89%) than ECF1 (68.7%). Despite the lower sensory scores of the enriched formulation, ECF1 remained acceptable to more than two‐thirds of the children.

Differences in sensory perception were even more pronounced among mothers. CCF consistently received high scores across all evaluated sensory attributes, including color, aroma, taste, texture, and overall acceptability, whereas ECF1 scored significantly lower, particularly for taste and aroma (*p* < 0.001). Consequently, CCF achieved a substantially higher overall Acceptability Index than ECF1, with 96.3% of mothers reporting an AI ≥ 70%, compared with only 31.7% for ECF1. The greater preference for CCF is likely explained by its similarity to traditional complementary porridges commonly consumed in South Kivu, whereas the incorporation of dried dagaa fish probably altered the flavor and aroma, resulting in lower maternal acceptance.

In addition to the sensory evaluation, the amount of porridge consumed by the children was assessed as an objective indicator of product acceptability (Figure 3). Consumption was significantly higher for the control complementary porridge (CCF) than for the enriched complementary porridge (ECF1) (*p* < 0.001). Overall, 61.0% of children consumed the entire serving of CCF, compared with 42.7% for ECF1. However, a greater proportion of children consumed less than one‐quarter of the ECF1 serving than of the CCF serving. Accordingly, the mean intake score was significantly higher for CCF (3.99 ± 1.51) than for ECF1 (3.35 ± 1.70) on the 5‐point consumption scale.

**FIGURE 3:**
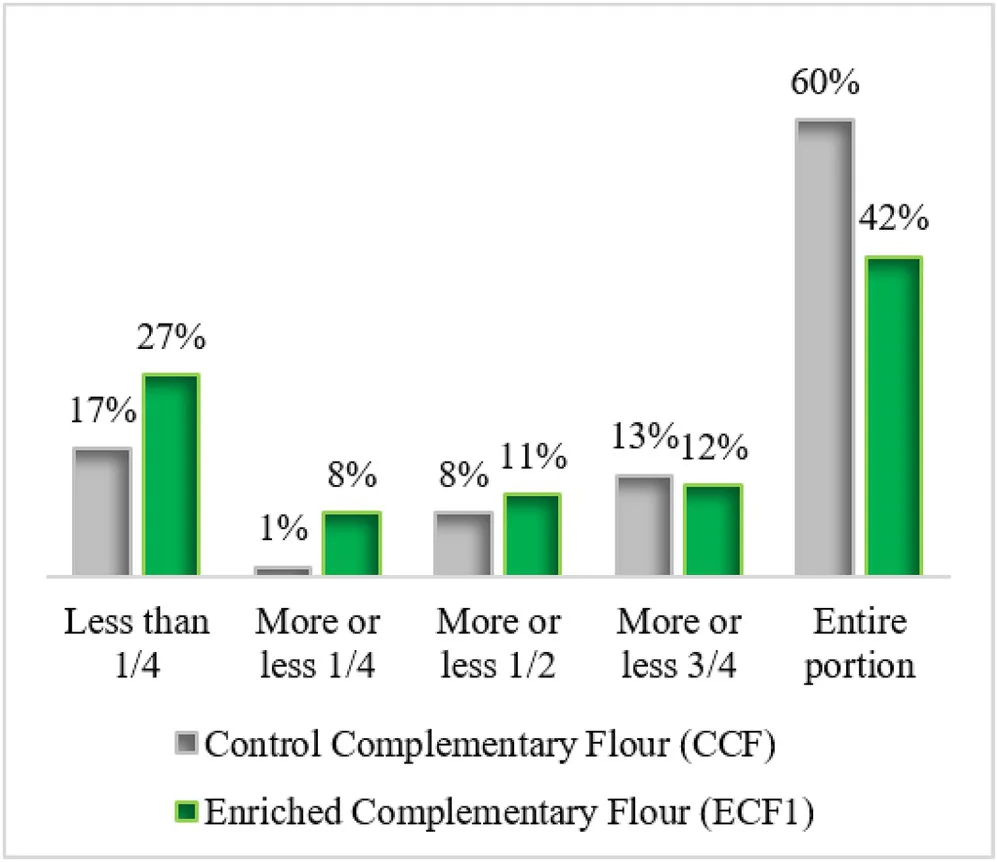
Estimating children's consumption.

Despite the lower intake of ECF1, nearly half of the children consumed the entire portion. These findings suggest that the sensory characteristics of porridges influenced children's consumption behavior. The paired *t*‐test confirmed this observation, showing a significant difference in intake scores between the two formulations (*t* = 3.28, *p* < 0.01).

Factors associated with porridge consumption were further investigated using multivariable linear regression models (Table 7). For the control complementary porridge, mothers' willingness to offer it to their children was the only factor significantly associated with the quantity consumed (*β* = 1.36, *p* = 0.023), whereas children's behavioral responses during feeding (alertness, calmness, or crying) were not significantly associated with intake. However, the model explained only a small proportion of the variability in consumption (*R* ^2^ = 0.05).

**TABLE 7:** Multivariate analysis of factors associated with porridge consumption.

	CCF			ECF1		
	*β* ± SE	*t*	*p*	*β* ± SE	*t*	*p*
Intercept	2.43 ± 0.97	2.50	0.015	1.23 ± 0.61	2.00	0.049
Child awake	0.42 ± 0.53	0.80	0.428	0.76 ± 0.65	1.16	0.251
Child calm	0.003 ± 0.79	0.003	0.997	0.11 ± 0.82	0.14	0.890
Child crying	−0.58 ± 0.90	−0.64	0.523	−0.079 ± 0.79	−0.10	0.920
Offer according to mother's acceptability	**1.36 ± 0.58**	2.33	**0.023**	**2.11 ± 0.32**	6.65	**< 0.001**

*Note:* The values are in bold because the *p*‐value is significant.

Abbreviations: *β*, regression coefficient; SE, standard error; *t* and *p*, associated statistics.

For the enriched complementary porridge (ECF1), maternal willingness to provide the product remained the strongest independent predictor of children's intake (*β* = 2.11, *p* < 0.001). As observed with CCF, children's behavioral responses were not significantly associated with consumption. The model explained 38% of the variability in intake (*R* ^2^ = 0.38), indicating that maternal acceptance played a substantially greater role in determining children's consumption of the enriched formulation than that of the traditional control porridge.

### 3.4 Storage Stability of the Formulated Complementary Flours

The proximate and selected mineral compositions of the control complementary flour (CCF) and the enriched complementary flour (ECF1) at baseline and after 6 months of ambient storage are shown in Table 8.

**TABLE 8:** Proximate and mineral composition of formulated complementary flours at baseline and after 6 months of ambient storage.

	Baseline		Endline	
	CCF	ECF1	CCF	ECF1
Moisture (%)	4.98	3.85	7.35	5.96
Ash (%)	2.03	5.48	1.43	5.03
Protein (%)	14.10	33.13	15.13	33.71
Lipid (%)	7.16	7.16	7.13	6.82
Iron (mg/100 g)	7.35	11.53	8.38	12.50
Zinc (mg/100 g)	2.23	6.46	2.97	6.22

*Note:* CCF: 50% maize, 25% sorghum and 25% soybean flour; ECF1: 30% maize, 10% sorghum, 20% soybean flour and 40% dried dagaa fish flour.

Moisture content increased in both formulations during storage, rising from 3.85 to 5.96 g/100 g in ECF1 and from 4.98 to 7.35 g/100 g in CCF. Despite this increase, moisture content remained below 10% in both flours, indicating favorable storage conditions. Similarly, protein content remained relatively stable throughout storage, increasing slightly from 33.13 to 33.71 g/100 g in ECF1 and from 14.10 to 15.13 g/100 g in CCF. Lipid content showed minimal variation over the storage period. A slight decrease was observed in ECF1 (7.16 to 6.82 g/100 g), whereas lipid content remained essentially unchanged in CCF (7.16 to 7.13 g/100 g). The mineral composition was also maintained during storage. Iron content increased slightly in both formulations, from 11.53 to 12.50 mg/100 g in ECF1 and from 7.35 to 8.38 mg/100 g in CCF. Zinc concentrations remained relatively stable, with only minor changes observed after storage (6.46 to 6.22 mg/100 g in ECF1 and 2.23 to 2.97 mg/100 g in CCF).

Microbiological analyses showed that both the control flour (CCF) and the enriched flour (ECF1) met microbiological safety standards throughout the storage period (Table 9). Coliforms, *E. coli*, and *Salmonella* spp. were not detected in either formulation at baseline (December 2022) or after 6 months of storage (June 2023). Yeast and mold counts increased slightly during storage, from 1.34 × 10^2^ to 2.20 × 10^2^ CFU/g in CCF and from 2.35 × 10^2^ to 4.40 × 10^2^ CFU/g in ECF1. However, these values remained well below the maximum acceptable limit of 10^4^ CFU/g, indicating satisfactory microbiological stability during storage.

**TABLE 9:** Microbiological quality of flours.

	Coliforms (CFU/g)	*Escherichia coli* (CFU/g)	Salmonella (CFU 25 g)	Yeasts and mold (CFU/g)
Methods	ISO 4832	ISO 7251	ISO 6785	ISO 21527
Limits	> 10^2^	10	Absent in 25 g (−)	> 10^4^
*December*				
CCF	(−)	(−)	(−)	1.34 × 10^2^
ECF1	(−)	(−)	(−)	2.35 × 10^2^
*June*				
CCF	(−)	(−)	(−)	2.2 × 10^2^
ECF1	(−)	(−)	(−)	4.4 × 10^2^

*Note:* CCF: 50% maize, 25% sorghum and 25% soybean flour; ECF1: 30% maize, 10% sorghum, 20% soybean flour and 40% dried dagaa fish flour; CFU/g: colony forming unit per gram.

## 4 Discussion

Appropriate complementary feeding is essential for preventing undernutrition and micronutrient deficiencies during the first 2 years of life. Micronutrient deficiencies, particularly of iron, zinc, and calcium, remain major public health concerns among infants and young children in low‐ and middle‐income countries (Belay et al. 2021; Dahdouh et al. 2019). Therefore, increasing the mineral density of complementary foods using locally available animal‐source ingredients is an important food‐based strategy to improve child nutrition. The present study developed and evaluated complementary foods enriched with dried dagaa, a locally available animal‐source food, to improve the nutritional quality of traditional cereal‐based porridges. Proximate composition analysis showed that incorporating dried dagaa markedly improved the nutritional quality of the complementary flours. Compared with the control formulation, ECF1 contained more than twice the protein content and substantially higher concentrations of calcium, iron, zinc, and ash. Similar increases in protein and micronutrient content following fish incorporation have been reported in Ghana, Ethiopia, and Malawi (Amankwah et al. 2009; Mekuria et al. 2021; Kaunda et al. 2025), highlighting the value of small fish as an affordable animal‐source ingredient for improving complementary foods. These improvements reflect the exceptional nutritional value of dagaa, which is naturally rich in highly digestible animal protein (72.1 g/100 g) and bioavailable minerals because it is consumed whole, including bones and viscera (Kawarazuka and Béné 2011; Thilsted et al. 2016; Byrd et al. 2021).

The energy, lipid, and moisture contents of the formulations also remained within or close to the ranges considered appropriate for complementary foods. Although the enriched formulations had slightly lower energy content than the control flour, their energy density remained close to the recommended level for complementary foods (WFP 2018). This reduction reflects the partial replacement of carbohydrate‐rich cereals with protein‐rich dried dagaa, thereby increasing nutrient density without compromising energy adequacy. Lipid contents were similar across formulations and remained within the recommended range (Lutter and Dewey 2003). The slightly lower fat content of ECF1 is attributable to the naturally low lipid content of dried dagaa and may also enhance storage stability by reducing the risk of lipid oxidation (Allen et al. 2006). All formulations had moisture contents below the recommended limits (Lutter and Dewey 2003; FAO/WHO 2023), indicating adequate processing and good storage stability, as low moisture reduces the risk of microbial growth and quality deterioration (Abdel‐Haleem and Omran 2014; Bhowmik et al. 2022). Mineral concentrations observed in ECF1 were higher than those reported for other fish‐enriched complementary foods (Adepeju et al. 2024), highlighting the nutritional value of dried dagaa as a locally available ingredient. Given the critical role of iron, zinc, and calcium in child growth, immune function, and the prevention of undernutrition (Brixi 2018), these findings suggest that dried dagaa enrichment could improve the nutritional quality of complementary foods and help address micronutrient deficiencies among young children in South Kivu. Although none of the formulations met the WFP (2018) recommendations for the 2 RNI levels of iron and zinc, the zinc content of the enriched formulations was within the range considered nutritionally beneficial. A 50 g serving of ECF1 provided a substantial proportion of the daily protein and mineral requirements for infants aged 6–12 months, particularly for zinc and magnesium, compared with the control formulation (FAO/WHO 2004). This nutritional improvement is largely attributable to the high nutrient content of dried dagaa. However, these estimates were based on total nutrient concentrations rather than on bioavailable nutrients. As phytates in cereals and legumes can reduce iron and zinc absorption, and neither phytate content nor mineral bioaccessibility was assessed, the actual physiological contribution of these minerals may be lower. Future studies should therefore assess mineral bio‐accessibility to better determine the nutritional value of these formulations.

Despite its superior nutritional quality, the enriched complementary food (ECF1) received lower sensory scores than the control formulation (CCF). Among mothers, the control porridge was consistently preferred for color, aroma, taste, texture, and overall acceptability. Similar findings have been reported in several African studies, in which fish‐enriched complementary foods were nutritionally superior but less preferred than conventional cereal porridges (Amankwah et al. 2009; Mekuria et al. 2021; Saka et al. 2018). This lower preference is likely related to the characteristic flavor and aroma of dried fish in porridges. Nevertheless, more than two‐thirds of mothers indicated that they would be willing to offer the enriched porridge to their children. This finding suggests that the lower sensory preference did not necessarily preclude potential use of the formulation.

Children also showed good overall acceptance of both porridges, although acceptability scores were higher for CCF than for ECF1. Because infants and young children cannot reliably express hedonic preferences, mothers assessed their children's acceptance by observing feeding behaviors, including willingness to eat, facial expressions, and food refusal. These scores should therefore be interpreted as proxy measures rather than direct assessments of children's preferences. Nevertheless, maternal proxy ratings are widely accepted in complementary feeding research and provide a practical way to evaluate food acceptability in this age group (Farrow et al. 2015; Kaunda et al. 2025).

Food intake assessment further supported the sensory findings. Children consumed significantly more of the control porridge than of the enriched formulation, reflecting the higher acceptability of CCF. However, nearly half of the children consumed the entire portion of enriched porridge, indicating that adding dried dagaa did not substantially reduce intake. The moderate acceptability of ECF1 may therefore partly reflect unfamiliarity with fish‐enriched porridge rather than intrinsic product rejection. Repeated exposure to unfamiliar foods during infancy has been associated with increased acceptance over time (Farrow et al. 2015). However, this mechanism was not directly evaluated in the present study and should therefore be considered a possible explanation rather than a demonstrated effect.

Maternal perception was also associated with children's food intake. In the present study, mothers' willingness to offer porridge was the only significant predictor of consumption. This finding suggests that caregiver perceptions may influence the practical use of nutrient‐dense complementary foods. Accordingly, product development may benefit from being accompanied by appropriate nutrition education and behavior‐change strategies, particularly when the sensory characteristics of an enriched product differ from those of familiar foods, emphasizing the need to combine improved food formulations with strategies to promote sustained use of nutrient‐rich complementary foods (UNICEF 2020).

The results suggest an inverse trade‐off between nutritional composition and sensory preference. Although ECF1 provided substantially higher concentrations of protein and minerals than CCF, it received lower sensory scores and was consumed in smaller quantities. This balance is particularly relevant for complementary foods, as a nutrient‐dense product may not be beneficial if its sensory characteristics substantially reduce consumption. Therefore, the findings support the need to consider both nutrient composition and acceptability when developing locally produced complementary foods.

Following the sensory evaluation, a second formulation (ECF2) with a lower proportion of dried dagaa was developed. Although ECF2 was not formally assessed using the hedonic protocol, it was successfully consumed throughout the subsequent 6‐month intervention trial. These preliminary observations support further investigation of formulations with lower fish inclusion levels. However, because ECF2 was not subjected to the same formal sensory evaluation, its acceptability cannot be directly compared with that of CCF and ECF1. Future studies should formally evaluate ECF2 and other optimized formulations using standardized sensory methods.

The storage study indicated that both complementary flours maintained satisfactory nutritional quality and microbiological safety throughout 6 months of storage. Although moisture content increased slightly, nutrient composition remained relatively stable, and no coliforms, *E. coli* , or *Salmonella* were detected. Yeast and mold counts also remained within recommended limits, indicating that roasting and appropriate storage conditions effectively preserved product quality. These findings are consistent with previous studies showing that roasting and low moisture content enhance the storage stability of complementary flours (Abdel‐Haleem and Omran 2014; Bauserman et al. 2015). Although the storage results were encouraging, several additional quality indicators warrant investigation. Lipid oxidation was not directly evaluated using peroxide value or thiobarbituric acid reactive substances (TBARS) analyses. Considering the relatively high proportion of fish in ECF1, future studies should include these measurements to provide a more comprehensive assessment of oxidative stability during storage. Likewise, aflatoxin contamination could not be determined before the feeding trial because analytical reagents were unavailable locally. Given young children's vulnerability to mycotoxin exposure, future research should systematically include aflatoxin screening to ensure compliance with Codex standards for complementary foods.

The main strength of this study lies in its integrated approach, which combines formulation, nutritional characterization, sensory evaluation, microbiological analyses, and storage stability within a single research framework and uses locally sourced ingredients.

Several limitations should be considered when interpreting the findings.

First, mineral bioavailability was not evaluated because in vitro digestion analyses could not be completed and phytate concentrations were not measured. Therefore, the estimated nutritional contribution from total mineral concentrations may not reflect actual absorption. Second, children's sensory acceptance was assessed using maternal proxy ratings rather than direct responses from the children. Thirdly, microbiological analyses were conducted only at baseline and after 6 months, limiting the ability to characterize changes in microbial counts between these two time points. Fourthly, the storage study was conducted for 6 months under a single set of ambient conditions, with no direct measurements of lipid oxidation or aflatoxin contamination. Finally, ECF2 was not formally evaluated using the sensory protocol. These limitations suggest areas for further investigation, rather than reducing the relevance of the nutritional and sensory findings observed in the present study.

## 5 Conclusion

This study demonstrates that enriching cereal‐ and legume‐based complementary flour with locally sourced dried dagaa substantially improved its nutritional composition, particularly its protein, iron, zinc, and calcium contents, compared with the control formulation. However, the enriched formulation received lower sensory scores and was consumed in smaller amounts than the control and traditional cereal porridge, indicating the need to balance nutritional enhancement with sensory acceptability. Despite these differences, both formulations were still widely accepted by children, and the enriched flour showed relatively limited changes in proximate composition and selected mineral contents over 6 months of ambient storage.

Overall, dried dagaa is a promising locally available ingredient for improving the nutritional quality of complementary foods. Further research should assess mineral bioavailability, optimize the sensory characteristics of fish‐enriched formulations, and evaluate longer‐term storage stability, including risks of oxidative and mycotoxin contamination. These steps are needed to strengthen the evidence for the potential use of dagaa‐based complementary foods in resource‐limited settings.

## Author Contributions

**Marie Amélie Nabuholo:** conceptualization, investigation, writing – original draft, funding acquisition, writing – review and editing, methodology, software, project administration, data curation, formal analysis, validation, resources. **Olouwafemi Mistourath Mama:** conceptualization, methodology, supervision, validation, writing – review and editing. **Adama Diouf:** writing – review and editing, visualization. **Esto Bahizire:** writing – review and editing, methodology, investigation, supervision. **Galibwa Kulimushi Alexis:** writing – review and editing, methodology, supervision. **Ciribagula Nkulwe Rodrigue:** writing – review and editing, methodology. **Nicole Idohou‐Dossou:** funding acquisition, writing – review and editing, validation, conceptualization, methodology, supervision, visualization, resources.

## Funding

This work was supported by a grant from the Nestlé Foundation.

## Ethics Statement

This study was conducted in accordance with the Declaration of Helsinki. The Ethics Committee of the Catholic University of Bukavu granted ethical approval (approval number UCB/CIES/NC/014/2023). Given the low literacy levels in the study area, oral informed consent was obtained from all participating mothers prior to enrolment after they received complete information about the study in the local language.

## Conflicts of Interest

The authors declare no conflicts of interest.

## Data Availability

The data that support the findings of this study are available on request due to privacy/ethical restrictions.
